# Supplementary material for: Unchanged but undecided: Reproductive intentions among Ghanaian women following COVID-19 lockdowns in two large metropolises in Ghana
Source: PLoS One. 2025 Aug 11;20(8):e0328298. doi: 10.1371/journal.pone.0328298 (PMC12338832; doi:10.1371/journal.pone.0328298)
Supplement: Attachment — Submitted filename: Response to Reviewers.docx [file pone.0328298.s002.docx]

PONE-D-24-25240

Unchanged but undecided: Reproductive intentions among Ghanaian women following COVID-19 lockdowns in two large metropolises in Ghana

PLOS ONE

Response to reviewers’ comments

We wish to express our profound gratitude to the reviewers for their comments. We strongly believe that our attempt to address the issues raised has greatly improved the quality of our manuscript. Below are point-by -point responses to each of the comments:

Please include the following items when submitting your revised manuscript:

Journal requirements:

1. Please ensure that your manuscript meets PLOS ONE's style requirements, including those for file naming.

**Response:** We have revised the manuscripts to meet PLOS ONE’s style requirements.

2. Please include a complete copy of PLOS’ questionnaire on inclusivity in global research in your revised manuscript.

**Response:** A copy of the PLOS’ questionnaire on inclusivity in global research has been uploaded as supporting information.

3. We note that the grant information you provided in the ‘Funding Information’ and ‘Financial Disclosure’ sections do not match.

When you resubmit, please ensure that you provide the correct grant numbers for the awards you received for your study in the ‘Funding Information’ section.

**Response**: The information provided in the ‘Funding Information’ section and the ‘Financial Disclosure’ section have been amended. There is however no grant number because the study was commissioned by the Gender Studies and Human Rights Documentation Centre with funding from INEVAWG.

4. Thank you for stating the following financial disclosure:
   This study was funded by International Network to End Violence against Women and Girls (INEVAWG).

Please state what role the funders took in the study. If the funders had no role, please state: "The funders had no role in study design, data collection and analysis, decision to publish, or preparation of the manuscript." 
If this statement is not correct you must amend it as needed. 
Please include this amended Role of Funder statement in your cover letter; we will change the online submission form on your behalf.

**Response**: We declare that the "The funders had no role in study design, data collection and analysis, decision to publish, or preparation of the manuscript."

5. Thank you for stating the following in the Acknowledgments Section of your manuscript:
   Many thanks to the International Network to End Violence Against Women and Girls (INEVAWG Network) for funding the study and the Gender Studies and Human Rights Documentation Centre for commissioning the study in Ghana. Thanks also to the Ghana Statistical Services, particularly, Mrs. Joyce Date and Mr. Isaac Dadson, our field research assistants, and other stakeholders without whom the study could not have been carried out.

Response: We have amended the acknowledgement and have removed the funding information from the statement.

We wish to mend the funding information to read: “this study was funded by the International Network to Violence against Women and Girls (INEVAWG), South Africa.

6. Please include your full ethics statement in the ‘Methods’ section of your manuscript file. In your statement, please include the full name of the IRB or ethics committee who approved or waived your study, as well as whether or not you obtained informed written or verbal consent. If consent was waived for your study, please include this information in your statement as well.

**Response**: We have included information on the ethics in the methods section in the manuscript. This includes the full name of the ethics committee and the number, as well as information on how consent was obtained. This section is marked “Ethical considerations” in the manuscript.

7. We note that you have indicated that there are restrictions to data sharing for this study. For studies involving human research participant data or other sensitive data, we encourage authors to share de-identified or anonymized data. However, when data cannot be publicly shared for ethical reasons, we allow authors to make their data sets available upon request. For information on unacceptable data access restrictions, please see <http://journals.plos.org/plosone/s/data-availability#loc-unacceptable-data-access-restrictions>.

Before we proceed with your manuscript, please address the following prompts:

a) If there are ethical or legal restrictions on sharing a de-identified data set, please explain them in detail (e.g., data contain potentially identifying or sensitive patient information, data are owned by a third-party organization, etc.) and who has imposed them (e.g., a Research Ethics Committee or Institutional Review Board, etc.). Please also provide contact information for a data access committee, ethics committee, or other institutional body to which data requests may be sent.

b) If there are no restrictions, please upload the minimal anonymized data set necessary to replicate your study findings to a stable, public repository and provide us with the relevant URLs, DOIs, or accession numbers. Please see <http://www.bmj.com/content/340/bmj.c181.long> for guidelines on how to de-identify and prepare clinical data for publication. For a list of recommended repositories, please see <https://journals.plos.org/plosone/s/recommended-repositories>. You also have the option of uploading the data as Supporting Information files, but we would recommend depositing data directly to a data repository if possible.

Please update your Data Availability statement in the submission form accordingly.

**Response**: The data for this study will be available on request. We shall deposit the data with the College of Humanities Ethics Committee. They can be reached by email: [ech@ug.edu.gh](mailto:ech@ug.edu.gh); and by phone: +233-303933866.

8. When completing the data availability statement of the submission form, you indicated that you will make your data available on acceptance. We strongly recommend all authors decide on a data sharing plan before acceptance, as the process can be lengthy and hold up publication timelines.

**Response**: This has been addressed above.

9. Please ensure that you refer to Figure 1 in your text as, if accepted, production will need this reference to link the reader to the figure.

**Response**: This has been done.

10. Please remove all personal information, ensure that the data shared are in accordance with participant consent, and re-upload a fully anonymized data set.

**Response:** all personal information has been removed from the document.

**Comments to the Author**

Reviewer #2: 
Abstract
1. The abstract mentions the potential influence of pre-existing social context but does not state the specific factors (e.g., economic status, education, cultural norms) may have influenced reproductive intentions.

**Response**: The abstract has been amended to “These effects interact with an individual’s social context, including socioeconomic status, cultural norms, family size and structure, to shape reproductive intentions”.
2. The abstract does not specify the research method and research design used in the study e.g. purely quantitative or mixed method. I suggest the authors should clarify this.

**Response**: The paper is part of a larger study where the mixed method approach was used to collect the data. However, for this paper, the quantitative aspect of the data was used. We have amended the abstract to indicate that the research design was quantitative. This has also been made clearer in the methods section.

3. The abstract does not state the specific statistical techniques used e.g. the multivariate model, logistic regression, multiple regression etc. which would help clarify the analysis.

**Response**: These statistical techniques have been specified in the abstract. The statistical technique used for Chi-square and Fisher’s exact tests.

4. The abstract mentions the need for future work to accommodate more ambiguity in characterizing reproductive intentions, it does not elaborate on the broader implications of these findings for policy or public health interventions.

**Response**: The broader implications of the study has been indicated in the abstract.

Introduction
1. The first sentence is incomplete ("met with a swift pandemic shook..."). This sentence needs to be revised for clarity and to ensure it flows logically.

**Response**: This has been amended.

2. The phrase "fertility intentions" is mentioned, it might be useful to explain it briefly earlier, particularly for readers who may not be familiar with the term.

**Response**: This has been elaborated. Fertility intention “can be understood as the desire, plan, or intention to have or to not have children”.

3. ……………” Some people were overworked, taking on additional childcare on top of remote jobs” What does this sentence mean? I suggest that the authors revise the sentence to make it clearer for readers to understand.

**Response**: This sentence has been revised.

4. While some studies are referenced, there is no explicit mention of the research gap this study addresses. The introduction could more clearly explain what is lacking in current literature and why this study is needed.

**Response**: this has been revised, and the gaps in the literature has been included.

5. This study sought to evaluate if and why Ghanaian women changed their reproductive intentions because of COVID-19 lockdown measures. This sentence is hanging. Please, check the sentence.

**Response**: This has been corrected.

Research methods
Research design
1. “This was an analytical cross-sectional study that used a convergent mixed methods design to primarily assess the economic and social impact of COVID-19 response measures on women and girls in Accra and Kumasi”…... Do you mean a "convergent mixed methods design"? If so, how is this design applied within a single study like this, particularly since the authors did not specify it in the abstract? This design is typically applicable in mixed-methods studies. I suggest the authors clarify this aspect to strengthen their explanation.

**Response**: The paper is part of a larger study which used mixed methods as the study design. This paper is based on the quantitative aspect of the study. The methods section has been revised to make this clearer.

2. How was this design applied in the study? The design appears inappropriate and, also seems isolated and not effectively integrated into the study. I suggest the authors thoroughly revise this section.

**Response**: The study design for the larger project was a mixed method, however, for this particular manuscript it is purely quantitative.

Study area
1. The choice of Accra and Kumasi is logical given their high case counts and economic significance, the justification would be stronger if the authors explicitly addressed issues of representation, regional comparisons, and broader applicability, it will strengthen the study setting.

**Response**: this has been revised. Data on teenage pregnancy and other reproductive health indicators in the two regions have been included to justify the choice of the study sites.

Recruitment of participants
1. Combination of sampling techniques. The random, systematic, and purposeful sampling techniques were used, the details of how these methods were combined are unclear.

**Response**: this has been made clearer in the methods section. The participants were purposively selected from different sectors of society to ensure representation.

2. Is this study quantitative or mixed-method? It seems like the study does not follow either approach.

**Response**: this is a purely quantitative study. The confusion has been cleared.

3. Additionally, the study does not provide details on how the different sampling methods (random, systematic, and purposeful) were applied in the data collection process.

**Response**: This has been addressed above.

4. “The exclusion criteria were non-residence and not being economically engaged in the study area for at least 6 months prior to initiation of COVID-19 lockdown and 6 months following the lockdown”. How were the participants excluded, and why were these participants excluded?"

**Response**: We thought that living in the community for at least 6 months prior to the lockdown and 6 months after the lockdown is enough time to measure for the effect of the environment on participants’ experiences.
5. The participants recruitment lacks clarity and justification. I suggest the authors should revise this section.

Response: this has been revised in the methods section.
Study tool
1. ‘Information was collected with ODK software using a 5-section structured questionnaire” What do you mean by ODK software and 5-section structured questionnaire?

**Response**: the ODK software is a free software used on android systems for data collection. As indicated above, the paper is part of a larger study where the questionnaire consisted of 5 sections. This paper is based on a section of the questionnaire.

2. How was the tool used to gather the data?

**Response**: the tool used to collect the data is the questionnaire.

3. Include how piloting improves the data collection process.

**Response**: Piloting the survey allowed the study team to refine wording, flow, and reliability of the questionnaire before implementing it with the study population.

Data analysis
1. There is no explanation of why Chi-squared and Fisher’s exact tests were chosen for hypothesis testing or why Firth’s logistic regression was used instead of other statistical techniques.

Response: “Fisher’s exact test was used when > 20% of expected cell counts was less than 5”
2. Chi-squared and Fisher's exact tests examine associations between categorical variables, but they do not account for confounders or control for multiple variables simultaneously. How did you account for or address confounding variables?

3. There is no clear explanation of how the hypothesis testing was conducted, including the null hypothesis, alternative hypothesis, and criteria for significance

Response: this has been indicated in the methods section on page 4.
Results
1. The presentation of the results is generally good; however, the authors should revise this section to avoid redundancy.

**Response**: This is done

Discussion.

1. The discussion is good but lacks implications for policy and practice. I suggest the authors include these aspects in the discussion.

**Response**: The discussion has been revised to include implications for policy and practice.

Conclusion
1. The conclusion does not mention how the findings can be applied to inform policy or practice, especially in areas such as family planning, healthcare services, or public health interventions.

**Response**: The conclusion has been revised to include the implication of the study.

2. There is no mention of the limitations of the study, which is a critical component of the conclusion.

**Response**: A subheading on the limitations has been included

3. The conclusion briefly touches on the need for future studies, but it lacks specific recommendations for further research in this area.

**Response**: Some recommendations have been added to the conclusion.

4. The conclusion references the COVID-19 lockdown, it doesn't connect the study's findings to broader trends or global implications.

Response: The conclusion has been revised to include broader global health implications.

Reviewer #3: 1. Though the study presents the Ghana case, it must give an insight into the global context on women reproductive intentions because of the Covid-19 lock down measures.
**Response**: Some literature on reproductive intentions in the global context have been provided. “A multi-country study that included data from 10,000 women across 30 countries found that around 14.4% reported changing intentions, which was comprised of 10% of women surveyed postponing pregnancy and 4.4% of women surveyed accelerating it".

2. Add 'sexual and reproductive healthcare' to the keywords

**Response**: ‘sexual and reproductive healthcare’ has been added to the keywords

3. Under the methods, describe the rigorous methods used in conducting the telephone interviews.

**Response**: The respondents were identified through the database of the Statistical Service for the business tracker study. The respondents were then contacted through the telephone to enquire about their availability and book an appointment for the interviews. Those who agreed to be interviewed were interviewed in-person.

4. There is the need to introduce some thick quotes from the qualitative data to support the quantitative analysis.

**Response**: The data for this paper is quantitative and not qualitative, so no quotes are available.

5. Break down the percentages for the 'other' in ethnicity and religion. Discuss scholarly the socio-demographics and how they could have potentially affected the results garnered.

**Response**: This has been included in the Table 1.

6. The concluding section is the weakest section. It has not presented any solid conclusions based on the study's results.

**Response**: The conclusion has been reviewed and made stronger.

7. Write in past tense as you are reporting on an already conducted study.

**Response**: The manuscript has been revised and the past tense used.
