# Supplementary material for: Unchanged but undecided: Reproductive intentions among Ghanaian women following COVID-19 lockdowns in two large metropolises in Ghana
Source: PLoS One. 2025 Aug 11;20(8):e0328298. doi: 10.1371/journal.pone.0328298 (PMC12338832; doi:10.1371/journal.pone.0328298)
Supplement: Attachment — Submitted filename: PLOS ONE-PONE-2 Response to reviewer comments.docx [file pone.0328298.s003.docx]

Dear Editor,

**RESPONSE TO REVIEWER COMMENTS**

We wish to thank you and all reviewers of our manuscript. We strongly believe that your suggestions for revision have greatly improved the current draft of the manuscript. Below are a point-by-point responses to all comments given:

Reviewer #1:

Good luck for this research publication. I have reviewed the revised version of the manuscript titled "Unchanged but undecided: Reproductive intentions among Ghanaian women following COVID-19 lockdowns in two large metropolises in Ghana" (Manuscript Number: PONE-D-24-25240R1).

The authors have adequately addressed the concerns and suggestions raised in the initial review. The revisions have strengthened the clarity and quality of the manuscript, and I believe it is now suitable for publication.

**Response: We wish to thank the reviewer for their scrutiny of the manuscript.**

Reviewer #2: The comments have been successfully addressed; however, there are a few remaining points for the authors to address.

1. Abstract.

The abstract is clear and comprehensive; however, the phrase 'quantitative research design' in line 28 is too broad. I suggest that the authors specify which type of quantitative research design was employed, such as descriptive quantitative research design, correlational quantitative research design, etc.

**Response: Thank you for pointing this out. The text has been updated to specify the research design as “correlational quantitative research design”. Please see line 28.**

2. Results.

“The prepositional phrase “of the”......is used frequently throughout the work (e.g., in line 28, in line 254, in line 328, etc.). I suggest that the authors vary the usage of this prepositional phrase to avoid monotony.

**Response: We thank the reviewer for the feedback. We have reviewed the entire manuscript and varied text previously containing the phrase to eliminate the phrase if not absolutely necessary.**

3. The limitations

The authors claimed in their response that a subheading on the limitations has been included. However, there is no subheading on limitations addressing the comment.

**Response: Thank you for your comment. Please see line 430 of the manuscript for the Strengths & Limitations subheading as previously submitted.**

Reviewer #3:

Thanks for revising the manuscript based on my review report. The revised version is much stronger and scholarly. However, since you clarified that the manuscript was from the quantitative aspect of a larger convergent parallel mixed methods study, I suggest you rewrite the methods section as such. Just state the broad research approach adopted for the study so you don't unnecessarily confuse readers. Also, give a more nuanced explanation of your choices of methods and their appropriateness for the study.

**Response: We wish to thank the reviewer for pointing out the possible confusion that readers may have with situating methods of the current study within that of the larger study from which the data was obtained. We have updated sections under study design and study population to eliminate possibly confusing aspects pertaining to the larger study and facilitate reader understanding of the current study. Please see lines 114-119; 140-148**
